# Long-Term Trends in Calcifying Plankton and pH in the North Sea

**DOI:** 10.1371/journal.pone.0061175

**Published:** 2013-05-01

**Authors:** Doug Beare, Abigail McQuatters-Gollop, Tessa van der Hammen, Marcel Machiels, Shwu Jiau Teoh, Jason M. Hall-Spencer

**Affiliations:** 1 Natural Resources Management, WorldFish, Batu Maung, Penang, Malaysia; 2 The Laboratory, Sir Alister Hardy Foundation for Ocean Science, Plymouth, Devon, United Kingdom; 3 Department of Fisheries, Institute for Marine Resources and Ecosystem Studies, IJmuiden, North Holland, The Netherlands; 4 Marine Biology and Ecology Research Centre, Plymouth University, Devon, United Kingdom; University of Gothenburg, Sweden

## Abstract

Relationships between six calcifying plankton groups and pH are explored in a highly biologically productive and data-rich area of the central North Sea using time-series datasets. The long-term trends show that abundances of foraminiferans, coccolithophores, and echinoderm larvae have risen over the last few decades while the abundances of bivalves and pteropods have declined. Despite good coverage of pH data for the study area there is uncertainty over the quality of this historical dataset; pH appears to have been declining since the mid 1990s but there was no statistical connection between the abundance of the calcifying plankton and the pH trends. If there are any effects of pH on calcifying plankton in the North Sea they appear to be masked by the combined effects of other climatic (e.g. temperature), chemical (nutrient concentrations) and biotic (predation) drivers. Certain calcified plankton have proliferated in the central North Sea, and are tolerant of changes in pH that have occurred since the 1950s but bivalve larvae and pteropods have declined. An improved monitoring programme is required as ocean acidification may be occurring at a rate that will exceed the environmental niches of numerous planktonic taxa, testing their capacities for acclimation and genetic adaptation.

## Introduction

Anthropogenic carbon dioxide emissions are changing ocean chemistry at a rate that is, as far as we know, unprecedented [1]. The ocean has absorbed around 30% of total CO_2_ emissions over the past 200 years causing a 30% increase in average surface ocean concentration of H^+^ causing a range of chemical changes known collectively as ocean acidification [2]. It is imperative that we understand the effects of ocean acidification on marine plankton; they are responsible for around half of global carbon fixation, they are a life history stage for most benthic organisms, and they form the basis of marine food webs, underpinning food security for millions of people worldwide [3].

It is currently unclear how present day chemical changes, caused by increasing CO_2_ levels in seawater, are affecting the plankton [4]. Experimental work shows that some marine microalgae are likely to grow well as *p*CO_2_ levels increase although they may have reduced nutritional quality with adverse ecological impacts on food webs via copepods to fish [5], [6].Calcifying taxa are widely predicted to be adversely affected, since ongoing acidification is rapidly lowering the calcium carbonate saturation state of surface waters, although work has begun on their ability to adapt or to evolve to cope [7], [8]. We know that there are large differences between responses of organisms to increasing levels of CO_2_ in seawater, even between strains of the same species [9]. Some laboratory studies have found that coccolithophores experience compromised lith formation when exposed to an acidified environment [10], while other work shows increased calcification in response to rising CO_2_ concentrations [11]. Responses to ocean acidification also vary depending upon food availability; mussels (*Mytilus edulis*) recruit from the plankton and grow well during periods of naturally high CO_2_ in the eutrophic conditions of the Baltic [12] but in oligotrophic conditions mollusc settlement is severely disrupted and adult mussel shells corrode as CO_2_ levels increase [13] since only well fed individuals can ‘afford’ the metabolic defenses that are needed to protect against increased acidity [14] . Parallels can be seen in the metabolic responses of other species to stress. Plaice (*Pleuronectes platessa*), for example, can cope with higher temperatures in areas of higher food availability [15].

Most investigations into the effects of decreasing pH on planktonic organisms have taken place in short-term laboratory, or mesocosm, experiments. Such work has provided detailed insights into the physiological effects of decreased pH, such as an ability of planktonic echinoderm larvae to maintain intracellular pH and calcify despite low extracellular pH [16], but very little information is available about the impacts that ocean acidification is having, or might have, on the abundance of calcifying plankton [17]. Studies at submarine volcanic vents with naturally high CO_2_ levels show that ocean acidification may radically alterfood webs [18] and models predict that marine plankton will soon experience pH conditions completely outside recent historical ranges [4]. Research, then, is urgently needed to determine whether or not ocean acidification will affect the ecology of this fundamentally important group of marine species.

**Figure 1:**
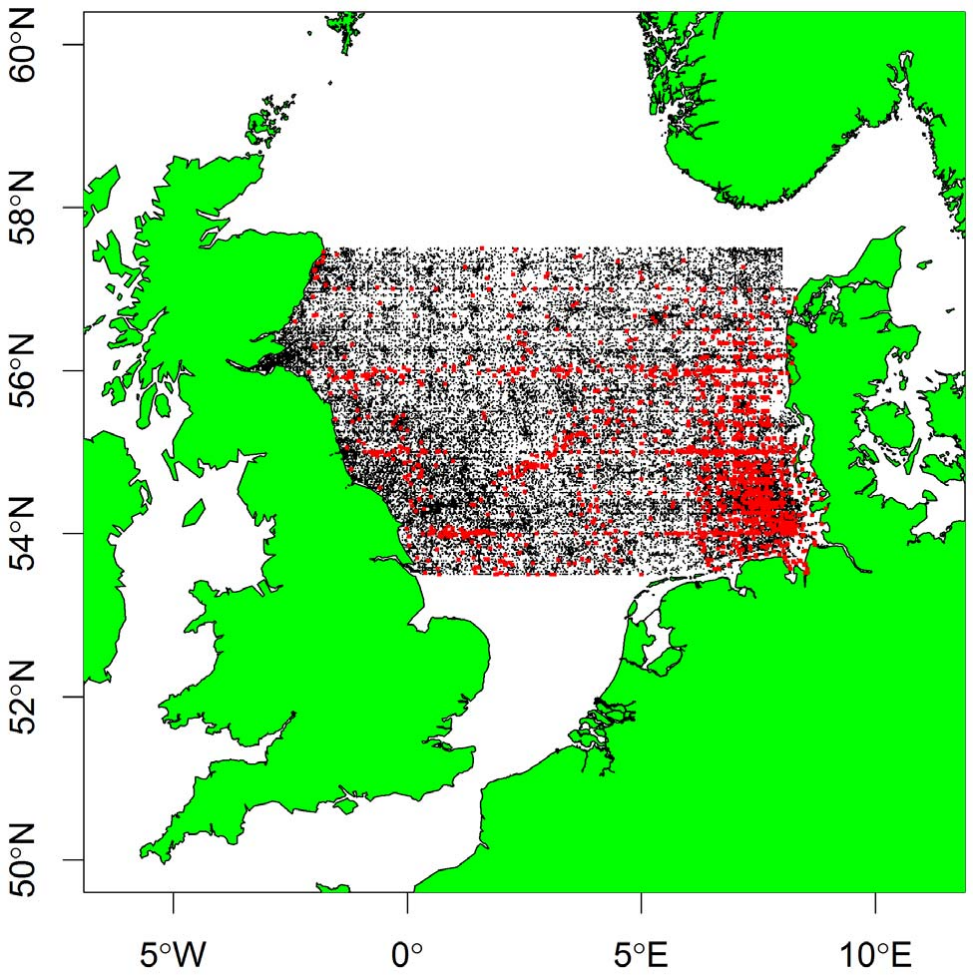
Area ‘4B’ in the North Sea based on a classification due to the International Council for the exploration of the Sea (ICES). Black dots in ‘4B’ are the sample locations for all the ICES oceanography data, while the red dots are the 6229 stations at which pH measurements were also done.

Continuous Plankton Recorder (CPR) survey data offers a long-term (>80 year) database of the abundance of plankton, in relation to environmental factors including pH, temperature and nutrients [19]. In the Northeast Atlantic, changes in the distribution of calcifying plankton during the past five decades are thought to be mainly driven by warming, although the influence of ocean acidification is unclear due to a paucity of data on changes in pH [see Figure S2 in [17]]. Here, we focus on changes in calcifying plankton taxa in a 280,000 km^2^ portion of the central North Sea (ICES area 4B; Figure 1) because this area has the richest pH dataset. This area contains the Dogger Bank, which is socioeconomically important as it supports extremely productive commercial fisheries, particularly for the planktivorous sandeel which themselves are an important food for larger fish, seabirds and cetaceans [20], [21]. We explore changes in the abundance of potentially vulnerable calcifying plankton groups, and their possible links to alterations in their environment, including pH. The purposes of this paper were to firstly assess trends in North Sea pH data and to determine whether there is any long-term relationship between the pH data and the abundance of calcifying plankton.

## Materials and Methods

### Oceanography data

Data on pH, and other oceanographic variables (e.g. sea temperature) were obtained from the International Council for the Exploration of the Sea (ICES). These data are freely available via the ICES website (www.ices.dk), and cover the entire North Atlantic and European continental shelf areas. In the North Atlantic there is very little historical pH data whereas the central North Sea (ICES area 4B; Figure 1) has the most numerous set of historical surface seawater pH records in the OSPAR region [17] (see Table 1) and for this reason was selected for further study. Despite the abundance of pH records for this region, there are difficulties with interpreting this historical dataset since metadata on how they were collected are unavailable [22]. ICES is now formulating plans to measure long term changes in pH, carbonate parameters and saturation states of aragonite and calcite in support of assessment of risks to and impacts on marine ecosystems, but at present the ICES pH database is the best available information for the region [22]. Our statistical analyses on the connection between the planktonic calcifiers and pH was only carried out on data between 1980 and 2010, since in the earlier period the pH data are too sparse.

**Table 1:** ICES oceanographic database: total numbers of observations in the North Sea 1958–2010.

ICES area	Location	Data points (stations sampled)	pH	Temperature
4A	Northern North Sea	151,305	1,685	149,447
4B	Middle North Sea	131,413	6,229	128,001
4C	Southern North Sea	27,902	3,318	25,902

### Plankton Data

The Continuous Plankton Recorder survey has collected ∼1 million plankton samples in the North Sea/North Atlantic during the last eight decades, and is operated by the Sir Alister Hardy Foundation for Ocean Science (SAHFOS) in Plymouth, UK. The survey, which routinely identifies ∼500 plankton taxa, most to species level, has had a virtually unchanged methodology since 1931, making it the world's longest and most spatially-extensive macro-ecological marine dataset (see [23] for more information on CPR methodology).

Phytoplankton cells are identified and recorded as either present or absent across 20 microscopic fields spanning each section of silk; CPR phytoplankton abundance must, therefore, be considered semiquantitative as each species is recorded once per field, independent of the number of cells in a field. Zooplankton analysis is carried out in two stages with small (<2 mm) zooplankton identified and counted on-silk and larger (>2 mm) zooplankton enumerated off the silk [23]. The CPR survey uses silk with a relatively large mesh size (270 µm), which is known to undersample most plankton taxa, in particular small-sized groups such as coccolithophores and foraminifera. Clogging in patches of high biomass plankton can reduce the effective mesh size of the CPR silk [24]. The proportion of individuals captured by the silk is, however, a roughly consistent fraction of the *in situ* abundance of each taxon, reflecting the major changes in abundance, distribution, and community composition of the plankton, and is consistent and comparable over time [24]. CPR data for the small coccolithophorid *Emiliania huxleyi,* for example, have been found to show interannual and spatial patterns in agreement with data derived from satellite remote sensing [25]. Therefore, CPR abundance data are best used to explore relative, rather than absolute, changes in the abundance and frequency of occurrence of plankton.

**Figure 2:**
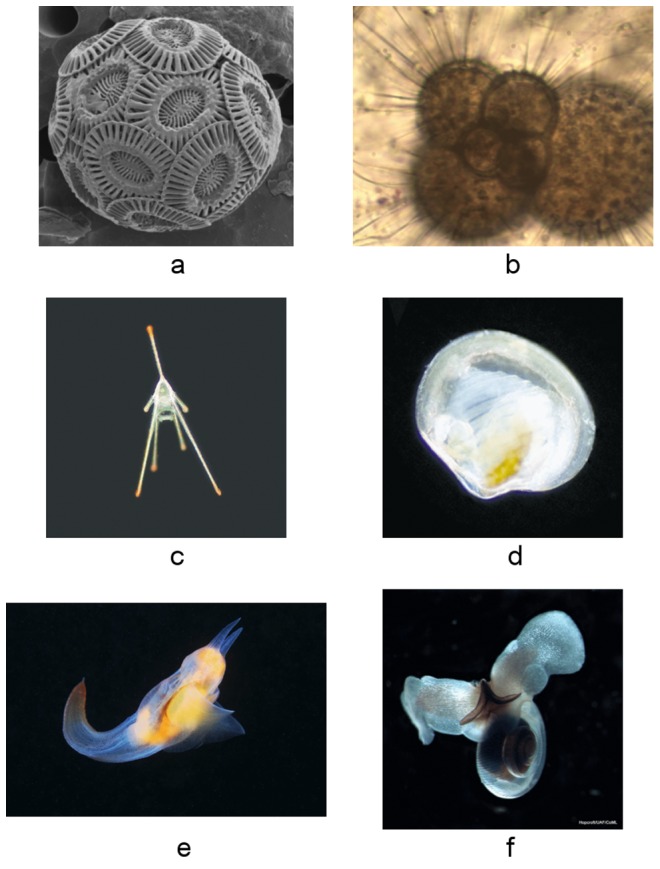
Calcifying plankton: (a) *Emiliania huxleyi* (Photo credit, G. Hallegraef); (b) Foraminiferan *Globerigina* spp. (Photo credit, J. Bijma); (c) *Echinocardium spp.* larvae (Photo credit, R. Kirby); (d) Bivalve larvae (Photo credit, R. Kirby); (e) *Clione limacina* (Photo credit, R. Hopcroft); and (f) *Limacina helicina* (Photo credit, R. Hopcroft).

CPR data for ICES area 4B for the following six calcifying taxa were obtained from SAHFOS: (i) coccolithophores; (ii) foramiferans; (iii) echinoderm larvae; (iv) bivalve larvae; (v) Clione limacina; and thecosomes (Figures 2a–f). Data were available as averages for ICES area 4B per month per year between 1958 and 2010 (see [26] for methodology). Abundances for Clione limacina, thecosomes., echinoderm larvae, and bivalve larvae reflect quantitative abundance; however, due to changes in their enumeration, the values for coccolithophores and foraminifera represent percent frequency of occurrence on CPR samples.

### Combining the data

The CPR data were available as averages per month per year for ICES area 4B between 1958 and 2010. The ICES oceanographic data were, however, sent to us in raw form (Figure 1) with information on the exact location (longitude and latitude) of each station together with the position in the water column of the actual sample, and its ‘sounding’ depth. In order to merge the datasets, therefore, monthly averages were also created for each oceanographic variable (eg. pH, temperature, and salinity) in the ICES dataset. Since CPR data are taken at the surface [27] only oceanographic data recorded in the top 25 m were used. Similarly, any data from very shallow areas (<10 m) were omitted from the analyses since these can be highly variable due to river inflows and changing salinity. To average pH data over a monthly time-period they were first converted to hydrogen ion concentrations, from which the pH could then be re-calculated. Once this new simpler dataset had been built, it was merged with the CPR plankton dataset, on which all subsequent analyses were based.

### Data exploration

**Figure 3:**
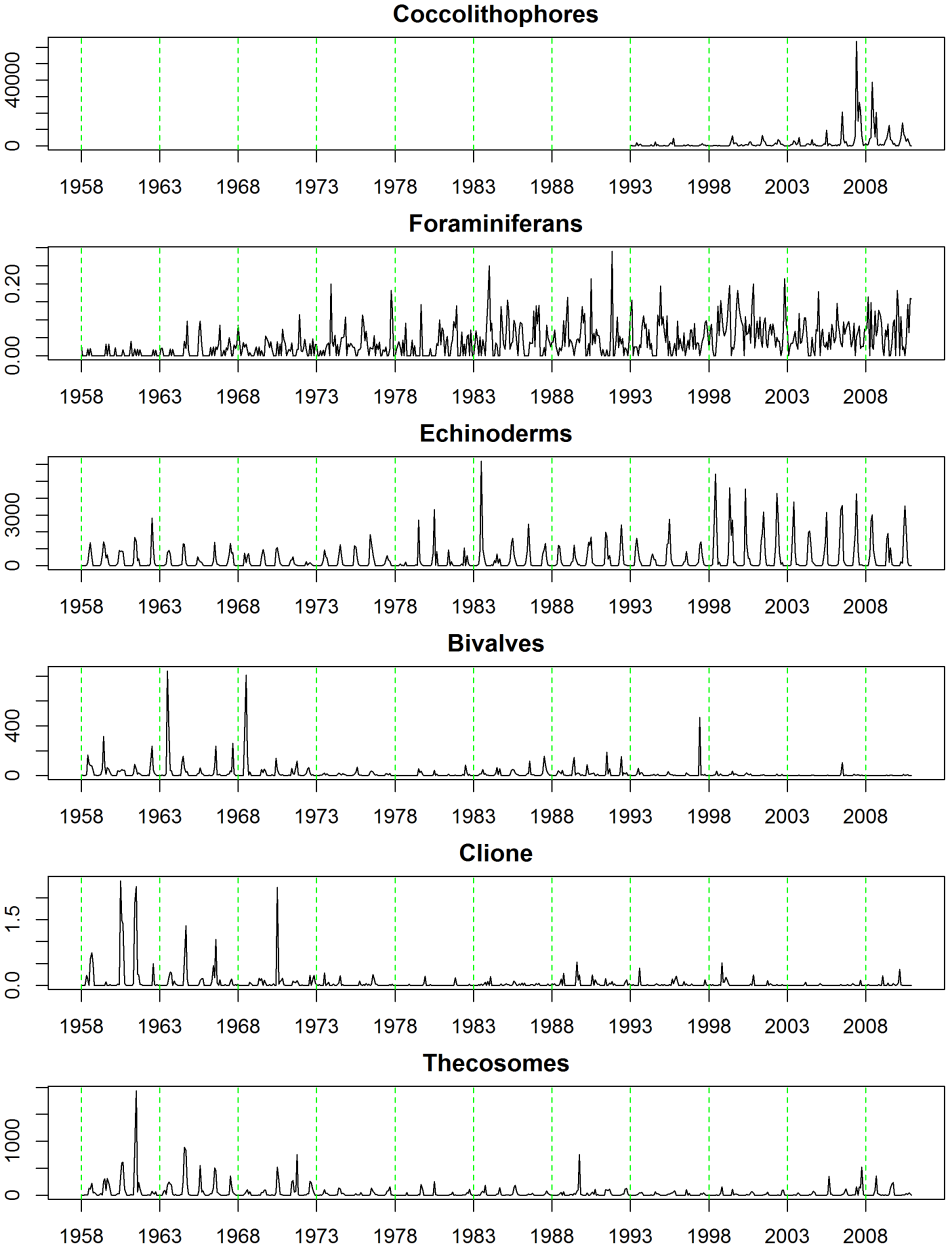
Long-term changes in the abundance of calcifiying plankton in ICES area ‘4B’ between 1958 and 2010.

**Figure 4:**
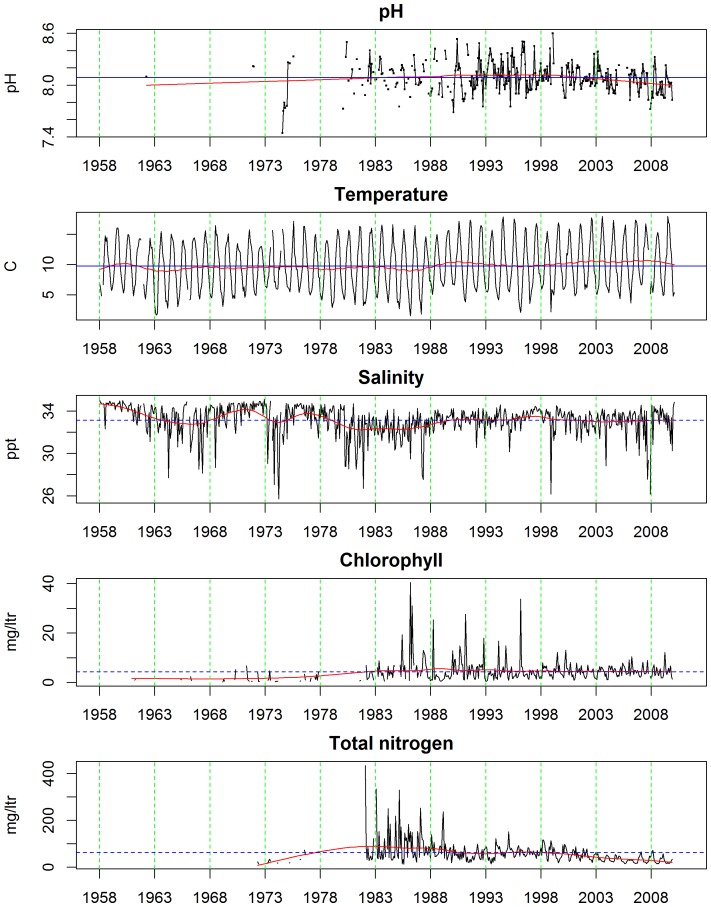
Long-term change in oceanographic variables between 1958 and 2010. Blue dotted line is the average and the red line is the long-term trend estimated using super-smoother.

**Figure 5:**
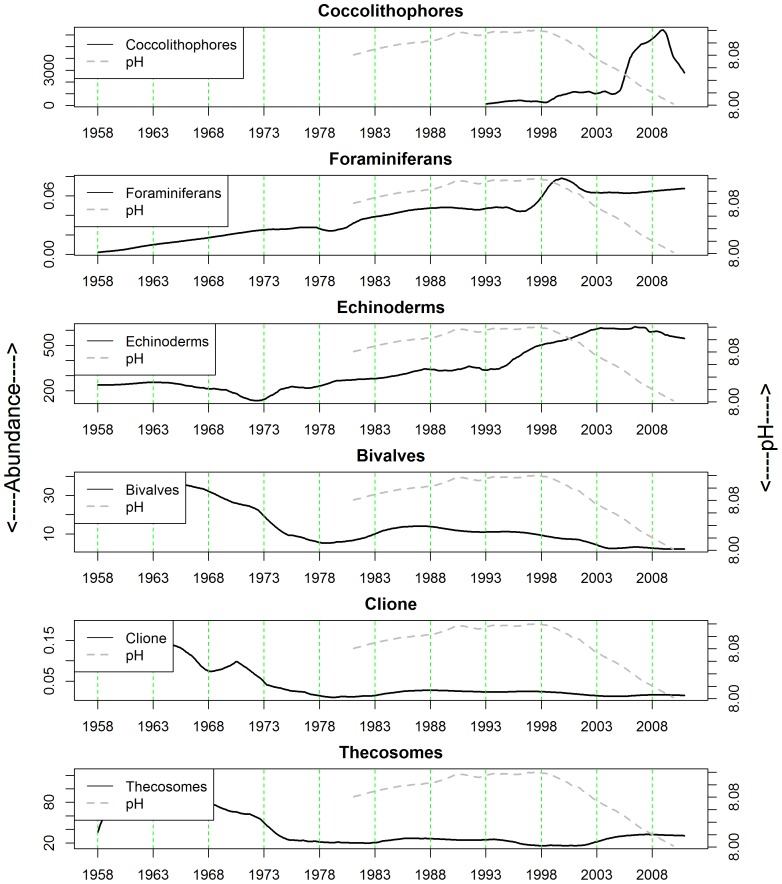
Long-term trends in the abundance of calcifying plankton between 1958 and 2010. The trends were estimated using Friedman's super-smoother in which abundance is modeled as a function of long-term trend.

Firstly a ‘long-term trend’ variable was calculated and appended to the dataset. This represents absolute time from January 1958; hence January 1958 is ‘1’ and January 1959 is ‘13’ and so on. Temporal changes in the groups of calcifying plankton, and oceanographic variables were first examined visually by simply plotting them as a function of this long-term trend (Figures 3 & 4). Subsequently, long-term changes in the abundances of each group or species were summarized using Friedman's ‘super smoother’ [28] and these are plotted in Figure 5. Super smoother is a running lines smoother which chooses between three spans for the lines. The best of the three smoothers is chosen by cross-validation for each prediction. The best spans are then smoothed by a running line smoother and the final prediction chosen by linear interpolation.

### Statistical modeling

All the time-series datasets examined here have obvious long-term trends and cyclical seasonal components, which means that they all tend to be correlated with each other, e.g. Table 2.

**Table 2:** Correlation coefficients between bivalve larvae, *Clione limacina*, thecosomes, pH and temperature.

Taxonomic group	pH	Temperature
Bivalvelarval_abundance	0.27	0.26
Clione abundance	0.07	0.17
Thecosome abundance	−0.09	0.32

These relationships can be misleading, and any results based on these pairs of observations could be spurious. It is well known that correlation can show an apparent relationship between two variables that have no causal link: the relationships existing because both data sets have underlying trends and seasonal variations. Any series that has a trend will tend to be correlated to any other series that has a trend. For this reason, it is preferable to quantify and describe the trends and seasonal effects first, before comparing multiple series. This is usually achieved by working with the residuals of a fitted time series model, which is what we do here. The time dependency in the pH, bivalve larvae, *Clione*, and thecosome data were modeled using harmonic seasonal regressions [29] in which both linear and quadratic long-term trends were assessed in the model selection process. The individual harmonic terms were selected using a standard stepwise approach. The residuals from each of these fitted models were then examined for any residual dependency between the datasets which might provide statistically more convincing evidence of causality. The data we used are appended to this publication as (Table S1) and are available online or directly from the authors.

## Results

Both the biological and oceanographic time-series are strongly seasonal (Figs 3 & 4) although seasonality in the foraminiferan category is the most irregular. In terms of long-term trend, the calcifying plankton can be divided into those with trends increasing (Figure 5) and those with trends falling (Figure 5). Those that proliferated are coccolithophores (Figure 2a), foraminifera (Figure 2b) and echinoderm larvae (Figure 2c), although it should be recalled that coccolithophore data were only available after 1992. Between 1958 and 1972 the abundance of echinoderm larvae in the CPR fell slightly, after which it has risen fairly steadily, with levels in 1983 and 1998 being especially high (Figs 3 & 5). The abundance of foraminiferans has risen throughout the entire time period (1958–2010) and coccolithophore abundance has also increased markedly between 1993 and 2010 (Figs 3 & 5). Bivalve larvae (Figure 2d), *Clione limacina* (Figure 2e), and thecosomes . (Figure 2f) have all declined in abundance with similar patterns of long-term trend in the central North Sea (see Figs 3 & 5) with relatively higher abundance between 1950 and the early 1970s, although peak years were different amongst these taxa. For example, bivalve larval peaks were noted in 1963 and 1968 whereas for thecosomes the peak year was 1961. In summary, profound changes were noted: high numbers of bivalve larvae, for example, were recorded in 1997 but otherwise densities have been very low. Between 2003 and 2010, no bivalve larvae have been recorded in the CPR samples for the majority of years.

Long-term trends and seasonalities for the oceanographic variables we investigated are plotted in Figure 4. The black solid lines are the raw data (i.e. monthly averages), the blue dotted lines denote the mean levels for the entire series, while the solid red lines represent ‘smooth’ functions describing the long-term trend. Between about 1980 and 1989 pH levels were about average (coincident with the blue dotted line), then they rose, being above average until around 2002 when they began to fall. Apart from a period in the very late 1950s and early 1960s sea surface temperatures were consistently lower than the average until *circa* 1988 since when they have been higher. Note that this increase in sea-temperature in 1988 coincides with a widely cited ‘regime shift’ in the ecology of the North Sea [30]–[31]. The salinity data are rather noisy in contrast, the seasonal patterns much less obvious, and characterized by periodic exceptionally low readings. Overall there is a suggestion that salinity levels in ICES 4B fell between 1958 and 1988 since when they have been rather stable (Figure 4). Like pH, the chlorophyll and total nitrogen concentration data were only available since the early 1980s (Figure 4). Both these datasets were clearly noisier in the 1980s and early 1990s. Overall average levels of chlorophyll have not changed much, whereas total nitrogen concentrations have fallen steadily since the early 1980s due to legislation imposed during the 1970s limiting the use of fertilizer in agriculture [32].

In Figure 5 the estimated long term trend for each of the calcifying plankton categories is plotted together with that for pH to facilitate visual comparisons. Unfortunately the pH data are sparse prior to 1980 which is when the more thorough comparisons among the time-series, perforce, began. It is clear that average pH records have not declined steadily between 1980 and 2010 in ICES area 4B. They rose from ∼8.08 in the early 1980s to peak at ∼8.12 in 1998, since when the recorded pH fell to ∼8.0 in 2010. Bivalve larvae, *Clione*, and thecosomes have long-term trends that appear to be only very broadly parallel with the long-term trends in pH records. [Note that echinoderm larvae and foraminiferans have been rising steadily since the late 1950s, see Figure 5]. For this reason it was decided that subsequent statistical time-series modeling analyses, in support of our hypothesis that, *“calcifying plankton will be adversely affected by falling pH*”, would only be sensible between bivalve larvae, *Clione*, thecosomes, and pH. The best models for pH, bivalve larvae abundance, *Clione* abundance, thecosome abundance in ICES area 4B are given by the following harmonic regression models, where *t* is absolute time:

1. pH = 7.53+0.003t–0.000006t^2^–0.11 cos(2πt/12)+0.06 cos(4πt/12)– 0.03 sin(8πt/12)
2. Ln(Bivalves) =  −8.09+0.051t–0.0001t^2^–1.08 sin(2πt/12)–1.82 cos(2πt/12)
3. Ln(Clione) =  −11.87+0.039t+0.00005t^2^–0.54 sin(2πt/12)+0.64 cos(2πt/12) –0.36 cos(4πt/12)
4. Ln(Thecosomes) =  5.5987−0.01780t+0.00002t^2^–2.3620 sin(2πt/12)−0.2878 sin(4πt/12)

**Figure 6:**
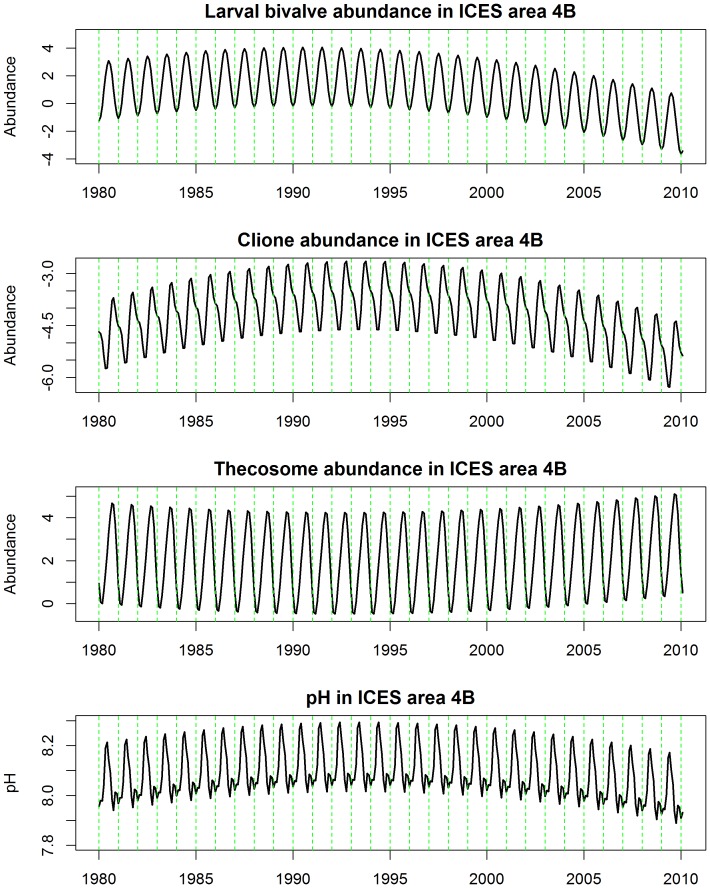
Predicted values from the harmonic seasonal regression models fitted to bivalves, *Clione*, thecosomes and pH (ie. output from models 1,2,3 & 4).

Model 1 is an ordinary linear model which fitted the pH data adequately but, due to the skewed nature of the bivalve, *Clione*, and thecosome (Models 2,3, & 4) data, and the high prevalence of zeros, Generalized Linear Models with quasipoisson link functions were preferred [33]. The three time-series models (i.e. 1–4) are plotted in Figure 6a. In all models a quadratic long-term trend term was selected (see models 1–4 & Figure 6a). The shape of this trend with peaks on the early and mid 1990s was similar for three of the series (models 1,2 & 4, see also Figure 6a) but the pattern summarizing long-term trend in the thecosomes was opposite, with peaks at each end of the series (Figure 6a).

**Figure 7:**
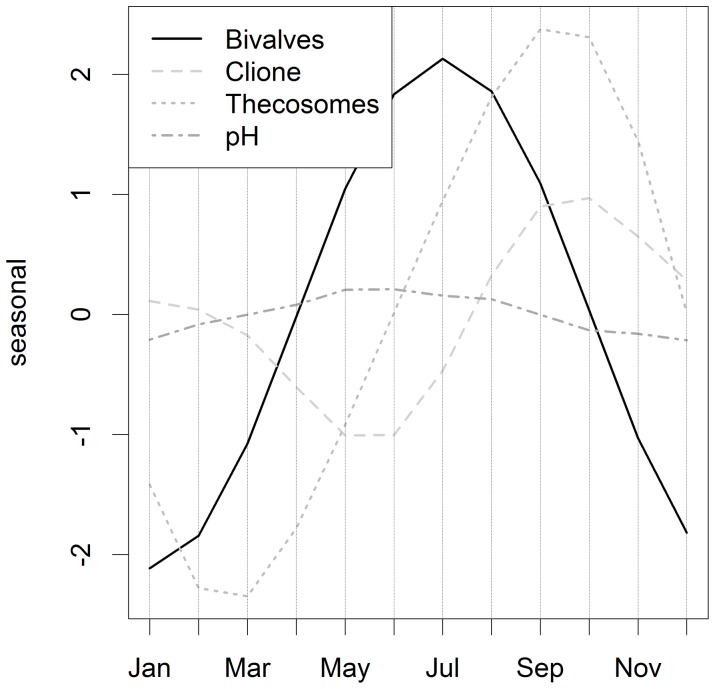
Seasonality in pH, bivalve, *Clione* and thecosome abundance in ICES area 4B (i.e. output from models 1,2,3 & 4).

The seasonalities were all quite different in shape. Average pH records peak in July (Figure 7) which happens because photosynthesizing phytoplankton are using CO_2_ to fix organic material for growth reducing dissolved CO_2_ levels which increases pH, or makes the water more alkaline. The abundance of planktonic bivalves was highest in July, while *Clione* and thecosome abundances peaked in October, and September respectively (Figure 7).

After trend and seasonal removal using these models the residuals from each were regressed against each other and the results are displayed in Tables 3a,b and c. It is clear that after ‘removing’, or accounting for, long-term trend and season there is no relationship between pH in ICES area 4B and either bivalve larvae, *Clione* or thecosome abundance. We also tried ‘Granger causality’ tests [34], too, which do not alter the conclusions.

**Table 3:** Analysis of variance tests.

**a. Response: residuals bivalve abundance**					
	*Df*	*Sum Sq*	*Mean Sq*	*F value*	*Pr (>F)*
*Residuals(pH)*	1	39.2	39.191	2.0778	0.1506 (n.s.)
*Residuals*	286	5394.6	18.862		
**b. Response: residuals *Clione* abundance**					
	*Df*	*Sum Sq*	*Mean Sq*	*F value*	*Pr (>F)*
*Residuals (pH)*	1	0.1424	0.142432	2.2847	0.1318 (n.s.)
*Residuals*	286	17.8295	0.062341		
***c.* Response: residuals thecosomes abundance**					
	*Df*	*Sum Sq*	*Mean Sq*	*F value*	*Pr (>F)*
*Residuals (pH)*	1	4.2	4.1968	0.1396	0.709 (n.s.)
*Residuals*	286	8598.5	30.06		

It should be noted that average pH may not, of course, be the most important factor; it may be either maximum or minimum pH (or other environmental drivers) or the variability around them that more realistically affect abundance of plankton. During our investigations we repeated the analyses described above by also calculating the minimum and maximum pH in ICES 4B, instead. After detrending and deseasonalising the data in the identical way as described above for the average pH, we found that the results were qualitatively identical, ie. no relationships were detected.

## Discussion

North Atlantic calcifying plankton distribution exhibited an abrupt shift in their distribution in the mid 1990s which has been attributed to climate-induced changes in temperature [17], although the effects of ocean acidification are unclear due to a lack of historical pH data for this region. Here we focus in on a pH data-rich region of the North Sea to more closely examine trends in distribution and abundance of calcified plankton. We found that average pH records have not been decreasing uniformly in the central North Sea despite steadily increasing levels of CO_2_ in the atmosphere [2]–[3]. This contrasts with the recent rapid rate of acidification reported in the southern North Sea [35] and at long-term monitoring stations at Hawaii, Tatoosh Island in the North Pacific, and Bermuda [36]–[37]. Since the mid to late 1990s, however, pH records have fallen, but whether or not this is the beginning of a more persistent long-term decline is not known. Given the level of concerns over ocean acidification, seawater carbonate monitoring stations are clearly urgently needed [22]. Although ICES sets out quality control guidelines in their guide to CTD data collection, metadata describing the details of how the pH is measured (details of pH meters, buffers etc.) are not yet collected by ICES. Modeling efforts are underway to attempt to fill data gaps in pH data, but in the North Sea model-derived pH and pCO_2_ remain uncertain, largely due to riverine input and primary production [38]. Because of these limitations, and in order to examine change in pH at a comparable regional spatial scale to plankton, we used monthly mean pH. It is worth noting here that a framework for international coordination of ocean acidification observations is currently being developed, involving the US National Oceanic and Atmospheric Administration (NOAA), the International Ocean Carbon Coordination Project (IOCCP) the Global Ocean Observing System (GOOS) and ICES which will improve the situation in future [22].

The abundances of three out of the six calcifying taxa that we identified as being potentially vulnerable to ocean acidification fell between 1958 and 2010, while the abundances of both coccolithophores and foraminiferans, by far the most important planktonic CaCO_3_ producers in the world ocean, are actually soaring in the North Sea (Fig. 5). *Clione* and thecosomes are thought to be highly susceptible to pH fluctuations as they produce aragonite skeletons. The abundance of both has fallen since the late 1950s, although thecosomes increased again slightly between 2002 and 2010 (Fig. 5). The only plankton groups whose dynamics have similar long-term trends to pH were *Clione*, bivalve larvae and thecosomes. There were, however, no confirmatory *statistical* relationships that we could find between, either the *Clione,* thecosomes. or the bivalve larval categories and pH. The quadratic trends between pH, bivalve larvae and *Clione* series are similarly shaped (Fig. 6a) and move in the same direction, while the trend for the thecosomes category is opposite, with peaks at each end and a dip during the middle period (Fig 6a). This suggests that they still *could* be related or connected. Evidence, however, in the form of the temporally mis-matched seasonalities (Fig. 6b) plus other scientific information makes this very unlikely in our opinion. In order to arrive at this conclusion we would also have to accept a positive relationship between pH, bivalve larvae, and *Clione* and a negative one between pH and thecosomes. A pteropod time-series from the North Pacific suggested an increase in *Clione* and a decrease in thecosomes; but the drivers behind the abundance and biomass trends of the two taxa in that region are unclear, and there is no evidence to suggest change in pH as a driver [39]. Pteropod time-series in the California Current and the Northwest Atlantic also show no clear trends [40]–[41]. We believe, instead, that the long-term trends these three North Sea taxa are responding more to the complex, combined impacts of other climatic, chemical and biotic drivers. Temperature is thought to be the main driver of changes in North Atlantic calcified plankton distribution [17], [42]; however, we found no clear statistical link between thecosomes, *Clione*, and temperature in the North Sea.

Larval surveys are a long established means of estimating the status of spawning stocks of many species of fish [43] and invertebrates [44]. Bivalve larvae fell in abundance from the 1950s to 2010 (Fig. 5) which Kirby and Beaugrand [45] argue is due to temperature-driven changes that have increased decapod predation on bivalves in the benthos, although ocean acidification could be involved. Overall increases of echinoderm larvae are also clear from the CPR data (Fig. 5) which Kirby et al. [46] attribute to rising temperatures. Although temperature certainly appears to be a major environmental factor driving changes in North Sea plankton, the contribution of changes in pH variability and decreasing saturation states of aragonite and calcite are as yet unknown. Increases in *p*CO_2_ adversely affect larval development in a wide range of marine molluscs [13] and it is this vulnerability that is thought to be causing failures in NE Pacific oyster production [47]. Conversely echinoderm larvae may be relatively robust, as despite having easily corroded high Mg-calcite skeletons, they can regulate their internal pH and continue larval calcification [16]. As some bivalves and echinoderms spend relatively short periods in the plankton the monthly time resolution of CPR data is not as good as, say, weekly or daily resolution and this will not expose the true degree of plankton variability. The series we examined, however, did reveal strongly seasonal cycles (see Fig 6b) with peaks in bivalve larval abundance seen in July, *Clione* abundance in October, and thecosome abundance in September.

Phytoplankton biomass is increasing in the North Atlantic basin [19] which the increased coccolithophore frequency of occurrence, revealed in the present study, reflects. The increase in phytobiomass is widely attributed to increases in temperature as a result of global climate change; coccolithophore blooms, in particular appear to respond to warm sea-surface temperatures [25] although the effects of increasing *p*CO_2_ levels may be stimulating algal growth [18].

It is thought that foraminiferan calcification rates will decrease with decreasing pH but experimental evidence currently exists for only 2 out of the >50 planktonic species known [9]. Laboratory experiments have shown that shell mass decreased in foraminiferans grown in an acidic environment [48] but shell formation and foraminiferan growth also depend on water temperature and food supply. Warming sea surface temperature as a result of climate changes could lead to increased foraminiferan growth rates [48], which may explain our observed trends in the North Sea CPR data. It is also possible that the calcified foraminiferans and coccolithophores have been able to proliferate in the North Sea through physiological acclimation and evolutionary adaptation to changes in carbonate chemistry [8].

In conclusion, we show that pH records have not fallen systematically in the central North Sea since the 1950s, although there has been a downward trend since the mid 1990s. There is little evidence to link the changes in pH records that have been recorded with calcified planktonic groups. Some calcified plankton have proliferated (echinoderm larvae, coccolithophores and foraminiferans) and so are able to cope with the variability in pH that has occurred in the central North Sea pH since the 1950s. Bivalve larvae and pteropods, however, have gone into decline; changes in seawater carbonate chemistry need to be monitored as there is concern is that ongoing ocean acidification may exceed the environmental niches of numerous planktonic taxa testing their capacities for acclimation and genetic adaptation.

## Supporting Information

Table S1 This is a comma-separated file, “s1.calcifiers.with.oceanography.csv” which contains all the data used in the analyses in the current manuscript. It comprises monthly averages of the SAHFOS/CPR plankton and ICES oceanographic data in area ‘4B’ each year. It has 18 columns which are as follows: month (the month sample was taken), year (the year sample was taken), Date (date format),Bivalves_abund (CPR bivalve larval abundance); Echinos_abund (CPR echinoderm larval abundance); Clione_abund (CPR clione abundance); Thecosomes_abund (CPR thecosomes abundance); Coccos_abund (CPR coccolithophore abundance); Forams_freq (CPR foraminiferan frequency); trend (absolute time in monthly blocks starting January 1958); ices_area (the ICES area); avph (average surface pH); avt (average sea surface temperature); avs (average surface salinity); avcphl (average surface chorophyll concentration); avntot (average surface concentration of total nitrogen), min.ph (the minimum surface pH recorded in that month, year combination in ICES area 4B); and max.ph (the maximum surface pH recorded in that month, year combination in ICES area 4B). Note that ‘surface’ means observations recorded between 0–20 m depth.
(ZIP)
